# Happiness and Satisfaction with Work Commute

**DOI:** 10.1007/s11205-012-0003-2

**Published:** 2012-02-14

**Authors:** Lars E. Olsson, Tommy Gärling, Dick Ettema, Margareta Friman, Satoshi Fujii

**Affiliations:** 1 Karlstad University, 651 88 Karlstad, Sweden; 2 University of Gothenburg, P.O. Box 500, 40530 Göteborg, Sweden; 3 Utrecht University, P.O. Box 80115, 3508 TC Utrecht, The Netherlands; 4 Kyoto University, C1-2-431, Katsura Nishikyo-ku, Kyoto, 615-8540 Japan

**Keywords:** Life satisfaction, Emotional well-being, Work commute

## Abstract

Research suggests that for many people happiness is being able to make the routines of everyday life work, such that positive feelings dominate over negative feelings resulting from daily hassles. In line with this, a survey of work commuters in the three largest urban areas of Sweden show that satisfaction with the work commute contributes to overall happiness. It is also found that feelings during the commutes are predominantly positive or neutral. Possible explanatory factors include desirable physical exercise from walking and biking, as well as that short commutes provide a buffer between the work and private spheres. For longer work commutes, social and entertainment activities either increase positive affects or counteract stress and boredom. Satisfaction with being employed in a recession may also spill over to positive experiences of work commutes.

**Electronic supplementary material:**

The online version of this article (doi:10.1007/s11205-012-0003-2) contains supplementary material, which is available to authorized users.

## Introduction

Everyday living in modern Western societies is dominated by mundane activities performed routinely, many motivated by obligations, although needs and desires are also common motives (e.g., Vilhelmson 1999; Michelson 2011; White and Dolan 2009). Since these activities constitute such a large part of everyday life, they are likely to have an impact on people’s overall life satisfaction and emotional well-being. This was recently demonstrated by Jakobsson Bergstad et al. (in press) for a subset of routine out-of-home activities. As a further indication of such an impact, research has shown that satisfaction with life domains including work or school, family life, and leisure (associated with performance of particular mundane routine activities) is positively correlated with overall life satisfaction (Pinquart and Silbereisen 2010).

Work commutes are in this context a neglected aspect of everyday life. A fact is still that billions of people commute to and from work every workday. An informal literature search of international transportation studies reveals that average commute times vary between 40 and 80 min, with public-transit taking longer than car commutes. An average of 4–10% of waking time on workdays is spent on commutes. Several previous, predominantly US studies have found that work commutes induce stress (see Novaco and Gonzales 2009, for review). Thus, it has been found that long work commutes in congested automobile traffic cause residual stress in the workplace (Novaco et al. 1990). Stress due to work commutes by public transit increases with the complexity of the commute (Wener et al. 2003) and with crowding in vehicles (Singer et al. 1978). In a similar vein, it has been argued that the negative effects of the length of work commutes substantially reduce the benefits of living in attractive places distant from the work place (Stutzer and Frey 2008).

In a study using the *day reconstruction method* to measure emotional well-being, Kahneman et al. (2004) found that the work commute belonged to the episodes that were most frequently associated with negative feelings during the day. The aim of the present study is to add to these research findings by investigating how overall life satisfaction and emotional well-being are affected by the work commute. Our approach is different in that satisfaction with the work commute is measured such that its correlation with overall life satisfaction and emotional well-being can be directly assessed. The analyzed data were collected in Sweden where, as in other European countries, the travel mode split is more even than in the US where driving is predominant. The results therefore also provide a contrast to the previous research in the US demonstrating stress effects.

Happiness (also commonly referred to as subjective well-being) has attracted a plethora of cross-disciplinary research in recent years (e.g., see reviews by Dolan et al. 2008; Lyubomirsky et al. 2005). In line with this research, we refer to happiness or subjective well-being as a higher-order construct consisting of a cognitive and two affective components (Busseri and Sadava 2011). The cognitive component consists of a judgment of life satisfaction (evaluations of life circumstances) that is commonly measured by reliable self-report rating scales, for instance the 5-item satisfaction with life scale (SWLS) (Diener et al. 1985; Pavot and Diener 1993; Slocum-Gori et al. 2009) which will be used in the present study.

The affective components of happiness include the positive and negative moods and emotional episodes that people experience. Several self-report methods have been devised to measure these affective components. A distinction is whether the methods are on-line such that they assess immediate affects (Stone et al. 1999) or retrospective and memory-based (Schwarz et al. 2009). The positive and negative affect scale (PANAS; Watson et al. 1988) has frequently been used either on-line to measure current mood or retrospectively to assess the frequency and intensity of affects for a specified timeframe. On this measure happiness increases with the frequency and intensity of positive affect (PA), including emotions such as joy and delight, and decreases with the frequency and intensity of negative affect (NA), including emotions such as anger and fear. A measure of emotional well-being (also referred to as the affect balance) is constructed by computing the difference between retrospective assessment of the frequency and/or intensity of positive and negative affect.

Other research has shown that affect is related to two dimensions, a pleasantness–unpleasantness dimension labelled *valence* and an active–passive dimension labelled *activation* (e.g., see the affect grid, Russell 2003). Diener and Lucas (2000) accordingly proposed that measures of the affective components of happiness should be based on a dimensional description varying in valence and activation. The Swedish core affect scale (SCAS; Västfjäll et al. 2002) that will be used in the present study is such a measure based on the affect grid.

Both the cognitive and affective components of happiness may be influenced by the work commute (Ettema et al. 2010). Even though the work commute generally have an intended positive outcome—and would therefore have a positive effect on the cognitive component of happiness—it may still be experienced as stressful. Thus, how commuters react affectively should also be important, whether they are predominantly stressed, relaxed, excited or bored. We have therefore developed the satisfaction with travel scale (STS) to measure a cognitive component and two affective components of the experience of any type of travel (Ettema et al. 2011; Jakobsson Bergstad et al. 2011). The STS that will be used in the present study thus has three components: a cognitive evaluation of the quality of travel, an affective evaluation of feelings during travel ranging from stressed to relaxed, and an affective evaluation of feelings during travel ranging from bored to excited.

## Method

The participants were 713 work commuters (41.7% male; age ranging from 20 to 65 with a mean of 41.2 years) living in the three largest urban areas of Sweden (Stockholm population 850,000; Göteborg population 510,000; Malmö population 395,000) (for detailed sample characteristics, see Table S1 in the supporting information available online). The participants answered a mail questionnaire that had three consecutive modules consisting of questions about the work commute, overall happiness, and sociodemographics.

To minimize memory distortions (Schwarz et al. 2009) the most recent normal commute to and from work was targeted in the questionnaire. In the first module the participants first reported the date, departure and arrival times, intermediate stops, and travel modes. On the basis of the self-reports of mode use, work commutes were classified as made by car if car was used for at least one leg of the commute^1^ (to work *n* = 269; from work *n* = 259), as made by public transit (PT) if PT was used for at least one leg and that no car was used for any other leg (to work *n* = 251; from work *n* = 254), and as commutes by slow modes if the commuters walked or biked all legs (to work *n* = 165; from work *n* = 164).

In the same module the STS was then administered to assess satisfaction with the commute to and from work, respectively. The STS consists of nine seven-point adjective scales; three measuring quality of travel (worked very poorly [−3] − worked very well [3]; very low standard [−3] − very high standard [3]; worst imaginable [−3] − best imaginable [3]), three measuring positive activation versus negative deactivation (very bored [−3] − very enthusiastic [3]; very fed up [−3] − very engaged [3]; very hurried [−3] − very relaxed [3])*,* and three measuring positive deactivation versus negative activation (very stressed [−3] − very calm [3]; very tired [−3] − very alert [3]; very worried [−3] − very confident [3]). The order between the ratings scales was counterbalanced.

In the second module the SWLS (with the time frame last month) was first administered. An average was computed for ratings of agreement on 1 (do not agree) –7 (fully agree) scales to the following five statements: in most ways my life is close to my ideal; the conditions of my life are excellent; I am satisfied with my life; So far I have received the important things I want in life, and; if I could live my life over, I would change almost nothing.

A measure of the affective components were thereafter obtained as self-report ratings of the frequency (never = 0; rarely = 1; sometimes = 2; often = 3; very often = 4) during the last month of experiencing three intensities (slightly = 1; moderately = 2; very = 3) of the six positive emotions *glad*, *active*, *joyful*, *awake*, *peppy*, and *pleased* and the six negative emotions *sad*, *passive, depressed*, *sleepy*, *dull*, and *displeased*. An affect-balance index was constructed by multiplying the ratings of frequency and intensity for each emotion, then summing with a positive sign for the positive emotions and with a negative sign for the negative emotions.

## Results

A composite measure of satisfaction with the work commute was formed by averaging across all nine STS scales.^2^,^3^ As Table 1 shows, on this measure daily commute time (from 10 to 180 min) reduces satisfaction with the work commute. Slow commute modes (walking and biking) also result in more satisfaction than car and public transit (Table 2). A multiple linear regression analysis reported in Table 3 reveals a significant negative effect of daily commute time and a significant positive effect of slow modes (walking/biking) versus public transit or driving. By dichotomizing the affective components of STS, Table 4 shows that positive or neutral feelings dominate during the work commute. The negative effects of daily commute time are also observed for the affective components.

Table 5 summarizes the results of multiple linear regression analyses with SWLS and affect balance as dependent variables, separately for the commutes to work and from work (the full results are provided as Table S2 in supporting information available online). As can be seen, satisfaction with the commutes to and from work directly influences the affect balance as well as SWLS, directly or indirectly through the affect balance. In previous studies (reviewed in Lyubomirsky et al. 2005), socio-demographic factors have accounted for approximately 10% of the variance in SWLS. The present figure is 14% that decreases to 7% when affect balance is partialled out. Satisfaction with the work commute accounts for an additional 2% of the variance in SWLS and an additional 11–12% of the variance in the affect balance.

## Discussion

The key finding of the reported survey is that satisfaction with the work commute has a substantial influence on overall happiness, particularly on the balance between positive and negative affect (Table 5). This influence would be negative for participants who are dissatisfied and positive for those who are satisfied with their work commutes. On average satisfaction is high (Table 1), thus a positive contribution is made to overall happiness. In addition the present study fails to show that work commutes are predominantly stressful (Table 4), as previous research has found (Novaco and Gonzales 2009), although negative feelings during the work commute increases with the length of the commute.

**Table 1:** Means (M) and standard deviations (SD) on a composite measure of satisfaction (STS) with the commutes to and from work related to commute time

	Short commute time (<20 min)			Medium-long commute time (20–35 min)			Long commute time (>35 min)		
	*n*	M	(SD)	*n*	M	(SD)	*n*	M	(SD)
Commute to work	228	1.0	(0.9)	259	0.9	(1.0)	226	0.6	(0.9)
Commute from work	204	1.0	(1.0)	245	0.9	(0.9)	264	0.6	(0.9)

**Table 2:** Means (M) and standard deviations (SD) on a composite measure of satisfaction (STS) with the commutes to and from work related to primary travel mode

	Primary travel mode								
	Car			Public transit			Walking/biking		
	*n*	M	(SD)	*n*	M	(SD)	*n*	M	(SD)
Commute to work	269	0.9	(1.0)	251	0.5	(0.8)	165	1.2	(0.9)
Commute from work	259	0.9	(1.0)	254	0.5	(0.8)	164	1.2	(0.9)

**Table 3:** Unstandardized regression coefficients (b), 95% confidence intervals (CI), and *t* and *p* values from multiple linear regression analyses with the dependent variables satisfaction (STS) with the commutes to and from work and the independent variables socio-demographics, travel mode, and commute time

	STS to work			STS from work		
*N*	713			713		
Mean	0.9			0.8		
SD	0.9			1.0		
Cronbach’s α	0.87			0.89		

	*b* ± CI	*t*	*p*	*b* ± CI	*t*	*p*
Middle (36–50 years [1]) versus low age (19–35 years [−1])	−0.03 ± 0.11	−0.52	.606	−0.02 ± 0.12	−0.31	.755
High (51–65 years [1]) versus low age (19–35 years [−1])	0.21 ± 0.10	3.83	<.001	0.13 ± 0.11	2.38	.018
Men (1) versus women (−1)	0.04 ± 0.07	1.18	.240	0.02 ± 0.07	0.56	.575
Cohabiting (yes [1] vs. no [−1])	0.01 ± 0.10	0.22	.824	0.02 ± 0.10	0.33	.741
Children (yes [1] vs. no [−1])	0.01 ± 0.08	0.30	.766	−0.05 ± 0.08	−1.15	.251
Education (years in school)	0.00 ± 0.02	0.16	.872	0.00 ± 0.02	0.23	.821
Weekly working hours (0–72)	0.00 ± 0.01	0.66	.509	−0.00 ± 0.01	−0.30	.768
Large (1) versus small urban area (−1)	0.00 ± 0.10	−0.02	.984	0.02 ± 0.11	0.41	.686
Medium (1) versus small (−1) urban area	0.02 ± 0.10	0.35	.727	−0.03 ± 0.10	−0.53	.596
High (1) versus low household income (−1)	0.04 ± 0.13	0.53	.598	0.05 ± 0.13	0.66	.510
Average (1) versus low household income (−1)	−0.02 ± 0.10	−0.35	.728	−0.02 ± 0.11	−0.35	.725
Walking/biking (1) versus public transit (−1)	0.29 ± 0.11	5.07	<.001	0.36 ± 0.12	6.01	<.001
Car (1) versus public transit (−1)	−0.05 ± 0.10	−0.92	.360	−0.03 ± 0.11	−0.59	.555
Commute time (minutes)	−0.01 ± 0.01	−3.02	.003	−0.01 ± 0.01	−3.10	.002
Full model	*R* ^2^ = .12, *F*(14, 615) = 6.08, *p* < .001			*R* ^2^ = .12, *F*(14, 606) = 6.06, *p* < .001		

Missing values were excluded pairwise

**Table 4:** Frequency of negative (e.g., stressed, tired) versus neutral or positive (e.g., relaxed, alert) affects during commutes to and from work related to daily commute time

		Short daily commute time (<40 min) *n* = 215		Medium-long daily commute time (40–70 min) *n* = 256		Long daily commute time (>70 min) *n* = 242	
		Commute to work		Commute to work		Commute to work	
		Stressed	Neutral or relaxed	Stressed	Neutral or relaxed	Stressed	Neutral or relaxed
Commute from work	Stressed	12	21	11	35	25	35
	Neutral or relaxed	19	163	27	183	20	162

		Tired	Neutral or alert	Tired	Neutral or alert	Tired	Neutral or alert
Commute from work	Tired	28	29	36	31	51	32
	Neutral or alert	27	131	42	147	45	114

**Table 5:** Percentage accounted for variance in affect balance and life-satisfaction judgments (SWLS) explained by socio-demographic variables and satisfaction (STS) with commutes to and from work

	Commute to work				Commute from work			
	Affect balance		SWLS		Affect balance		SWLS	
	Model 1	Model 2	Model 1	Model 2	Model 1	Model 2	Model 1	Model 2
Affect balance	–	–	–	28	–	–		28
SWLS	–	28	–	–	–	28	–	–
Socio-demographic variables	9	3	14	7	9	3	14	7
STS to work	12	5	8	2				
STS from work					11	5	7	2
Total	21	36	22	36	20	36	21	36

The entries in the table are increments (∆*R* ^2^) in hierarchical regression analyses. All of the increments and the full models are statistically significant at *p* < .01 or less. The full results are given as Table S2 in supporting information available online

The present results add to previous findings by suggesting that affect associated with mundane routine activities in different life domains may play important roles for overall happiness. In fact, the role of satisfaction with the work commute is of the magnitude observed for several of the mundane routine activities investigated by Jakobsson Bergstad et al. (in press). We assume here that the causal direction is from satisfaction with the work commute to overall happiness. A reverse direction is however also conceivable. For instance, given the negative effects that unemployment has on overall happiness in the current economic situation, it is possible that the happiness derived from having a job spills over to the satisfaction with the work commute.

Why are the present results inconsistent with those of studies of work commuting, predominantly by car, in the US (Novaco and Gonzales 2009)? One factor is that biking and walking, more common in the present study conducted in Sweden (and this would be the same in several other European countries, e.g., The Netherlands), contributes more to satisfaction with the work commute than driving and public transit. That walking and biking provide desirable physical exercise is a reason for their popularity (Lawrence et al. 2006). If commutes are short, as walking and biking commutes usually are, they may also be appreciated as a buffer between the work and private spheres (Jain and Lyons 2008). Particularly over longer distances, satisfaction with the work commute decreases. It is an open question whether public transit leads to more satisfaction than driving when the length of the commute increases. Speaking for public transit is that, more than solo driving does, it allows for engagement in activities such as talking to others, resulting in PAs, and work or entertainment activities that may reduce stress and boredom.

Other possible factors accounting for satisfaction with the work commute are not directly related to the travel mode per se. Some research has demonstrated adaptation to adverse conditions (Frederick and Loewenstein 1999). The results of the present study do not exclude that some people report positive experiences because they adapt to negative effects of their work commute. These people may be susceptible to adaptation costs (e.g., physiological stress reactions Ng et al. 2009) which the self-report measures in the present study do not fully capture.

The findings in the present and other studies (Jakobsson Bergstad et al. in press, 2011) that experiences of work commutes and other mundane routine activities has measurable effects on overall happiness should be a reminder of that engagement in particularly meaningful activities such as practicing generosity, developing social relations or learning to manage stress (Lyubomirsky 2008), even though important, are not the only routes to happiness in life. For many people, being able to make the routines of everyday life work, such that positive feelings dominate over negative feelings resulting from daily hassles, may be equally important for their overall happiness. This insight is particularly significant to convey to policy makers who are responsible for spending tax money to improve municipality facilities.

## Electronic supplementary material

Below is the link to the electronic supplementary material.

Supplementary material 1 (DOC 191 kb)

## Footnotes

1 A commute to work may for instance have three trip legs: walking to the garage at home, driving the car to a parking lot close to the workplace, and walking from the parking lot to the workplace. This is classified as a car commute. If in the example public transit is used instead of driving, this is classified as a commute by public transit. The commute is classified as made by slow modes if the commuter only walks or uses a bicycle.

2 The correlations between the three components exceeded *r* ≈ 0.50, and the components’ correlations with the other measures were similar.

3 The indices are somewhat skewed, although within an acceptable range (SWLS = −0.72, affect balance = −0.50, STS to work = 0.11, and STS from work = 0.21). Comparisons between arithmetic means in Tables 1 and 2 are still valid and these central measures were preferred to distribution-free measures such as the medians having larger standard errors.
