# Supplementary material for: Happiness and Satisfaction with Work Commute
Source: Soc Indic Res. 2012 Feb 14;111(1):255–63. doi: 10.1007/s11205-012-0003-2 (PMC3560964; doi:10.1007/s11205-012-0003-2)
Supplement: Supplementary file 1 — Supplementary material 1 (DOC 191 kb) [file 11205_2012_3_MOESM1_ESM.doc]

Table S1. Sample characteristic.

|  | |  | **Urban area** |  |
| --- | --- | --- | --- | --- |
|  | | **Stockholm**  **(large)** | **Göteborg**  **(medium)** | **Malmö**  **(small)** |
| **Population** | | 850,000 | 510,000 | 295,000 |
|  | |  |  |  |
| **Sample size (n)** | | 246 | 258 | 209 |
|  | |  |  |  |
| **Response rate (%)** | | 22.8 | 23.7 | 22.8 |
|  | |  |  |  |
| **Women (%)** | | 56.1 | 59.7 | 57.9 |
|  | Missing | 0.0 | 0.8 | 1.0 |
|  |  |  |  |  |
| **Mean age (M/SD)** | | 41.8/12.9 | 40.9/13.1 | 40.8/12.7 |
|  | Missing (%) | 1.6 | 2.8 | 2.9 |
|  | |  |  |  |
| **Household type (%)** | |  |  |  |
|  | Single households without children | 21.5 | 26.4 | 21.1 |
|  | Single households with children | 7.7 | 3.1 | 6.2 |
|  | Cohabiting households without children | 39.0 | 31.0 | 35.4 |
|  | Cohabiting households with children | 30.5 | 39.1 | 36.4 |
|  | Missing | 1.2 | 0.4 | 1.0 |
|  | |  |  |  |
| **Number of years of education (M/SD)** | | 15.1/3.4 | 15.0/3.2 | 15.0/3.5 |
|  | Missing (%) | 1.9 | 1.6 | 1.9 |
|  | |  |  |  |
| **Working hours/week or more (M/SD)** | | 37.7/12.5 | 37.0/12.5 | 35.5/12.3 |
|  | Missing (%) | 0.8 | 2.4 | 5.3 |
|  | |  |  |  |
| **Employment type (%)** | |  |  |  |
|  | Employed | 76.4 | 80.1 | 76.1 |
|  | Unemployed | 2.3 | 0.8 | 1.4 |
|  | Own business | 4.3 | 6.9 | 6.2 |
|  | Student | 12.0 | 8.5 | 11.0 |
|  | Pensioner | 0.4 | 0.4 | 1.9 |
|  | Homemaker | 0.4 | 0.0 | 1.0 |
|  | Other | 3.9 | 2.0 | 1.0 |
|  | Missing | 0.4 | 1.3 | 1.4 |
|  | |  |  |  |
| **Monthly household gross income in ’000 SEK (%)** | |  |  |  |
|  | <42 | 40.3 | 35.4 | 36.4 |
|  | 42–64 | 34.9 | 32.9 | 31.1 |
|  | >64 | 17.4 | 26.0 | 19.6 |
|  | Don’t know | 4.7 | 2.8 | 7.7 |
|  | Missing | 2.7 | 2.8 | 5.3 |
|  |  |  |  |  |
| **Driver’s license (%)** | | 79.7 | 88.4 | 85.2 |
|  | Missing | 1.6 | 2.0 | 4.3 |
|  |  |  |  |  |
| **Access to a car (%)** | |  |  |  |
|  | Always | 59.3 | 53.7 | 62.7 |
|  | Several times a week | 14.7 | 11.0 | 13.4 |
|  | Once a week | 4.3 | 2.0 | 5.3 |
|  | Less than once a week | 9.3 | 13.0 | 6.2 |
|  | Never | 11.2 | 17.5 | 10.0 |
|  | Missing | 1.2 | 2.8 | 2.4 |

Table S2a. Unstandardized regression coefficients (b), 95% confidence intervals (CI), and *t* and *p* values from OLS multiple linear regression analyses with affect balance^a^ and SWLS^b^ as dependent variables and SWLS or affect balance, socio-demographics, and satisfaction with (STS) with the *commute to work* entered in steps as independent variables.

|  | **Affect balance** | | | **Affect balance** | | | **SWLS** | | | **SWLS** | | |
| --- | --- | --- | --- | --- | --- | --- | --- | --- | --- | --- | --- | --- |
| N | 713 | | | 713 | | | 713 | | | 713 | | |
| Mean |  | | |  | | | 5.0^a^ | | |  | | |
| Sum | 46.9 | | |  | | |  | | |  | | |
| Standard deviation | 38.2 | | |  | | | 1.3 | | |  | | |
| Cronbach’s α | .88^c^ | | |  | | | .91 | | |  | | |
|  | b±CI | t | p | b±CI | t | p | b±CI | t | p | b±CI | t | p |
| Affect balance |  |  |  |  |  |  |  |  |  | 0.018±0.002 | 15.66 | <.001 |
| SWLS |  |  |  | 15.48±1.94 | 15.66 | <.001 |  |  |  |  |  |  |
| **Increment (∆R^2^)** |  | | | **∆R^2^ = .28,**  ***F*(1, 628) = 245.13, *p*<.001** | | |  | | | **∆R^2^ = .28,**  ***F*(1, 628) = 245.13, *p*<.001** | | |
| Affect balance |  |  |  |  |  |  |  |  |  | 0.016±0.002 | 14.06 | <.001 |
| SWLS |  |  |  | 14.80±2.06 | 14.06 | <.001 |  |  |  |  |  |  |
| Middle (36–50 yrs [1]) vs. low age (19–35 yrs [-1]) | 1.12±4.70 | 0.47 | .636 | 3.32±4.07 | 1.60 | .110 | -0.15±0.15 | -1.88 | .061 | -0.17±0.13 | -2.43 | .016 |
| High (51–65 yrs [1]) vs. low age (19–35 yrs [-1]) | 0.52±4.36 | 0.23 | .816 | -0.87±3.78 | -0.45 | .652 | 0.09±0.15 | 1.27 | .204 | 0.09±0.12 | 1.33 | .184 |
| Man (1) vs. woman (-1) | -1.17±3.01 | -0.77 | .444 | -0.03±2.61 | -0.02 | .984 | -0.08±0.10 | -1.52 | .129 | -0.06±0.09 | -1.31 | .191 |
| Cohabiting (yes [1] vs. no [-1]) | -0.31±4.05 | -0.15 | .881 | -3.51±3.53 | -1.95 | .052 | 0.22±0.13 | 3.17 | .002 | 0.22±0.12 | 3.73 | <.001 |
| Children (yes [1] vs. no[-1]) | 0.01±3.37 | 0.01 | .996 | -0.42±2.94 | -0.28 | .777 | 0.03±0.11 | 0.51 | .609 | 0.03±0.10 | 0.58 | .559 |
| Years in school | 0.05±0.90 | 0.12 | .907 | 0.36±0.77 | 0.90 | .369 | -0.02±0.03 | -1.36 | .176 | -0.02±0.03 | -1.62 | .105 |
| Weekly working hours (0–72) | 0.47±0.25 | 3.65 | <.001 | 0.29±0.22 | 2.62 | .009 | 0.01±0.01 | 2.74 | .006 | 0.00±0.01 | 1.07 | .286 |
| Large (1) vs. small urban area (-1) | -2.59±4.11 | -1.25 | .213 | -0.42±3.57 | -0.23 | .817 | -0.15±0.13 | -2.12 | .034 | -0.10±0.12 | -1.73 | .084 |
| Medium-size (1) vs. small (-1) urban area | -0.94±4.06 | -0.46 | .646 | -0.53±3.51 | -0.30 | .766 | -0.03±0.13 | -0.41 | .685 | -0.01±0.12 | -0.21 | .837 |
| High(1) vs. low household income (-1) | 10.55±5.38 | 3.88 | <.001 | 4.22±4.74 | 1.75 | .081 | 0.43±0.18 | 4.72 | <.001 | 0.26±0.15 | 3.19 | .002 |
| Average(1) vs. low household income (-1) | -0.65±4.34 | -0.29 | .769 | 0.51±3.76 | 0.27 | .791 | -0.08±0.14 | -1.07 | .286 | -0.07±0.12 | -1.06 | .290 |
| **Increment** | **∆R^2^ = .09,**  ***F*(11, 618) = 5.50, *p*<.001** | | | **∆R^2^ = .03,**  ***F*(11, 617) = 2.40, *p*=.006** | | | **∆R^2^ = .14,**  ***F*(11, 618) = 9.05, *p*<.001** | | | **∆R^2^ = .07,**  ***F*(11, 617) = 5.77, *p*<.001** | | |

Table S2a (continued)

|  | b±CI | t | p | b±CI | t | p | b(±CI) | t | p | b±CI | t | p |
| --- | --- | --- | --- | --- | --- | --- | --- | --- | --- | --- | --- | --- |
| Affect balance |  |  |  |  |  |  |  |  |  | 0.015±0.002 | 11.86 | <.001 |
| SWLS |  |  |  | 12.68±2.09 | 11.86 | <.001 |  |  |  |  |  |  |
| Middle (36–50 yrs [1]) vs. low age (19–35 yrs [-1]) | 1.36±4.36 | 0.61 | .541 | 3.14±3.95 | 1.56 | .119 | -0.14±0.15 | -1.85 | .064 | -0.16±0.14 | -2.35 | .019 |
| High (51–65 yrs [1]) vs. low age (19–35 yrs [-1]) | -2.70±4.15 | -1.28 | .201 | -2.73±3.74 | -1.43 | .152 | 0.00±0.14 | 0.03 | .975 | 0.04±0.13 | 0.65 | .518 |
| Man (1) vs. woman (-1) | -2.54±2.86 | -1.75 | .081 | -1.15±2.59 | -0.88 | .382 | -0.11±0.10 | -2.21 | .028 | -0.07±0.09 | -1.60 | .109 |
| Cohabiting (yes [1] vs. no [-1]) | -0.52±3.77 | -0.27 | .787 | -3.18±3.44 | -1.82 | .069 | 0.21±0.13 | 3.21 | .001 | 0.22±0.11 | 3.69 | <.001 |
| Children (yes [1] vs. no[-1]) | -0.72±3.17 | -0.45 | .654 | -0.89±2.86 | -0.61 | .542 | 0.01±0.11 | 0.24 | .812 | 0.02±0.10 | 0.48 | .633 |
| Years in school | 0.11±0.84 | 0.26 | .796 | 0.36±0.76 | 0.93 | .355 | -0.02±0.03 | -1.34 | .182 | -0.02±0.03 | -1.61 | .109 |
| Weekly work hours (0–40) | 0.42±0.23 | 3.51 | <.001 | 0.29±0.21 | 2.64 | .008 | 0.01±0.01 | 2.57 | .010 | 0.00±0.01 | 1.16 | .247 |
| Large (1) vs. small urban area (-1) | -0.81±4.00 | -0.40 | .690 | 0.63±3.61 | 0.34 | .735 | -0.11±0.14 | -1.63 | .103 | -0.10±0.12 | -1.62 | .106 |
| Medium-size (1) vs. small (-1) urban area | -1.77±3.78 | -0.92 | .360 | -1.19±3.42 | -0.68 | .494 | -0.05±0.12 | -0.69 | .492 | -0.02±0.12 | -0.32 | .746 |
| High(1) vs. low household income (-1) | 9.86±5.04 | 3.84 | <.001 | 4.69±4.63 | 1.99 | .047 | 0.41±0.17 | 4.67 | <.001 | 0.26±0.16 | 3.29 | .001 |
| Average(1) vs. low household income (-1) | -0.77±4.04 | -0.37 | .710 | 0.20±3.65 | 0.11 | .914 | -0.08±0.13 | -1.09 | .278 | -0.07±0.12 | -1.03 | .305 |
| Walking/biking (1) vs. public transit (-1) | 0.06±4.45 | 0.03 | .978 | 0.70±4.02 | 0.34 | .733 | -0.05±0.15 | -0.65 | .517 | -0.05±0.14 | -0.73 | .465 |
| Car (1) vs. public transit (-1) | 1.95±4.12 | 0.93 | .353 | 1.25±3.72 | 0.66 | .508 | 0.06±0.14 | -0.77 | .443 | 0.03±0.12 | 0.40 | .686 |
| Daily commute time to work (minutes) | -0.05±0.17 | -0.64 | .522 | -0.06±0.15 | -0.79 | .432 | 0.00±0.01 | 0.16 | .873 | 0.00±0.01 | 0.48 | .629 |
| STS commute to work | 14.01±3.06 | 9.01 | <.001 | 8.87±2.89 | 6.04 | <.001 | 0.41±0.10 | 7.65 | <.001 | 0.20±0.10 | 3.91 | <.001 |
| **Increment** | **∆R^2^ = .12,**  ***F*(4, 614) = 23.63, *p*<.001** | | | **∆R^2^ = .05,**  ***F*(4, 613) = 11.41, *p*<.001** | | | **∆R^2^ = .08,**  ***F*(4, 614) = 15.49, *p*<.001** | | | **∆R^2^ = .02,**  ***F*(4, 613) = 3.84, *p*<.001** | | |
| **Full model** | **R^2^= .21,**  ***F*(15, 614) = 10.93, *p*<.001** | | | **R^2^= .36,**  ***F*(16, 613) = 21.37, *p*<.001** | | | **R^2^= .22,**  ***F*(15, 614) = 11.39, *p*<.001** | | | **R^2^= .36,**  ***F*(16, 613) = 21.90, *p*<.001** | | |

Note. Missing values were excluded pairwise.

^a^An index calculated as the sum of rated frequencies (*0=never; 1=rarely; 2=sometimes; 3=often; 4=very often*) multiplied by intensities (*slightly=1; moderately=2*; *very=3*) for negative emotions (*sad, passive, depressed, sleepy, dull,* and *displeased*) subtracted from the sum of rated frequencies multiplied by intensity for positive emotions (*glad, active, joyful, awake, peppy,* and *pleased*). The index ranges from -144 to 144.

^b^Average ratings of agreement (from 1 “do not agree” to 7 “fully agree”) to the following statements: *In most ways my life is close to my ideal; The conditions of my life are excellent; I am satisfied with my life; So far I have received the important things I want in life*; *If I could live my life over, I would change almost nothing*.

Table S2b. Unstandardized regression coefficients (b), 95% confidence intervals (CI), and *t* and *p* values from multiple linear regression analyses with affect balance^a^ and SWLS^b^ as dependent variables and SWLS or affect balance, socio-demographics, and satisfaction (STS) with the *commute from work* entered in steps as independent variables.

|  | **Affect balance** | | | **Affect balance** | | | **SWLS** | | | **SWLS** | | |
| --- | --- | --- | --- | --- | --- | --- | --- | --- | --- | --- | --- | --- |
| N | 713 | | | 713 | | | 713 | | | 713 | | |
| Mean |  | | |  | | | 5.0^a^ | | |  | | |
| Sum | 46.9 | | |  | | |  | | |  | | |
| Standard deviation | 38.2 | | |  | | | 1.3 | | |  | | |
| Cronbach’s α | .88 | | |  | | | .91 | | |  | | |
|  | b±CI | t | p | b±CI | t | p | b±CI | t | p | b±CI | t | p |
| Affect balance |  |  |  |  |  |  |  |  |  | 0.018±0.002 | 15.54 | <.001 |
| SWLS |  |  |  | 15.48±1.95 | 15.44 | <.001 |  |  |  |  |  |  |
| **Increment (∆R^2^)** |  | | | **∆R^2^ = .28,**  ***F*(1, 619) = 241.62, *p*<.001** | | |  | | | **∆R^2^ = .28,**  ***F*(1, 619) = 241.62, *p*<.001** | | |
| Affect balance |  |  |  |  |  |  |  |  |  | 0.016±0.002 | 13.96 | <.001 |
| SWLS |  |  |  | 14.80±2.08 | 13.96 | <.001 |  |  |  |  |  |  |
| Middle (36–50 yrs [1]) vs. low age (19–35 yrs [-1]) | 1.12±4.70 | 0.47 | .639 | 3.32±4.10 | 1.59 | .112 | -0.15±0.16 | -1.87 | .062 | -0.17±0.13 | -2.41 | .016 |
| High (51–65 yrs [1]) vs. low age (19–35 yrs [-1]) | 0.52±4.36 | 0.23 | .817 | -0.87±3.81 | -0.45 | .654 | 0.09±0.15 | 1.26 | .207 | 0.09±0.12 | 1.32 | .187 |
| Man (1) vs. woman (-1) | -1.17±3.01 | -0.76 | .448 | -0.03±2.63 | -0.02 | .984 | -0.08±0.10 | -1.51 | .132 | -0.06±0.09 | -1.30 | .194 |
| Cohabiting (yes [1] vs. no [-1]) | -0.31±4.05 | -0.15 | .881 | -3.51±3.55 | -1.94 | .053 | 0.22±0.13 | 3.15 | .002 | 0.22±0.12 | 3.70 | <.001 |
| Children (yes [1] vs. no[-1]) | 0.01±3.37 | 0.01 | .996 | -0.42±2.94 | -0.28 | .779 | 0.03±0.11 | 0.51 | .612 | 0.03±0.10 | 0.58 | .562 |
| Years in school | 0.05±0.90 | 0.12 | .908 | 0.36±0.78 | 0.89 | .372 | -0.02±0.03 | -1.35 | .179 | -0.02±0.03 | -1.61 | .108 |
| Weekly working hours (0–72) | 0.47±0.25 | 3.62 | <.001 | 0.29±0.22 | 2.60 | .009 | 0.01±0.01 | 2.72 | .007 | 0.00±0.01 | 1.06 | .289 |
| Large (1) vs. small urban area (-1) | -2.59±4.11 | -1.24 | .216 | -0.42±3.59 | -0.23 | .818 | -0.15±0.13 | -2.11 | .036 | -0.10±0.12 | -1.72 | .087 |
| Medium-size (1) vs. small (-1) urban area | -0.94±4.06 | -0.46 | .648 | -0.53±3.54 | -0.30 | .767 | -0.03±0.13 | -0.40 | .687 | -0.01±0.12 | -0.21 | .838 |
| High (1) vs. low household income(-1) | 10.55±5.38 | 3.85 | <.001 | 4.22±4.77 | 1.74 | .083 | 0.43±0.18 | 4.69 | <.001 | 0.26±0.15 | 3.17 | .002 |
| Average (1) vs. low household income (-1) | -0.65±4.34 | -0.29 | .770 | 0.51±3.79 | 0.26 | .792 | -0.08±0.14 | -1.06 | .290 | -0.07±0.12 | -1.05 | .294 |
| **Increment** | **∆R^2^ = .09,**  ***F*(11, 609) = 5.42, *p*<.001** | | | **∆R^2^ = .03,**  ***F*(11, 608) = 2.37, *p*=.005** | | | **∆R^2^ = .14,**  ***F*(11, 609) = 8.92, *p*<.001** | | | **∆R^2^ = .07,**  ***F*(11, 608) = 5.68, *p*<.001** | | |

Table S2b (continued)

|  | **Affect balance** | | | **Affect balance** | | | **SWLS** | | | **SWLS** | | |
| --- | --- | --- | --- | --- | --- | --- | --- | --- | --- | --- | --- | --- |
|  | b±CI | t | p | b±CI | t | p | b±CI | t | p | b±CI | t | p |
| Affect balance |  |  |  |  |  |  |  |  |  | 0.015±0.002 | 11.93 | <.001 |
| SWLS |  |  |  | 12.83±2.11 | 11.93 | <.001 |  |  |  |  |  |  |
| Middle (36–50 yrs [1]) vs. low age (19–35 yrs [-1]) | 1.20±4.41 | 0.53 | .595 | 3.07±3.99 | 1.51 | .131 | -0.15±0.15 | -1.91 | .057 | -0.16±0.14 | -2.38 | .018 |
| High (51–65 yrs [1]) vs. low age (19–35 yrs [-1]) | -1.11±4.16 | -0.52 | .601 | -1.63±3.74 | -0.85 | .395 | 0.04±0.14 | 0.56 | .577 | 0.06±0.12 | 0.87 | .383 |
| Man (1) vs. woman (-1) | -2.24±2.88 | -1.53 | .127 | -0.92±2.60 | -0.70 | .487 | -0.10±0.10 | -2.06 | .040 | -0.07±0.09 | -1.55 | .123 |
| Cohabiting (yes [1] vs. no [-1]) | -0.51±3.81 | -0.26 | .795 | -3.22±3.47 | -1.82 | .069 | 0.21±0.13 | 3.19 | .002 | 0.22±0.12 | 3.67 | <.001 |
| Children (yes [1] vs. no[-1]) | 0.37±3.20 | 0.22 | .823 | -0.16±2.89 | -0.11 | .916 | 0.04±0.11 | 0.73 | .465 | 0.04±0.10 | 0.70 | .482 |
| Years in school | 0.02±0.85 | -0.04 | .967 | 0.28±0.77 | 0.73 | .468 | -0.02±0.03 | -1.41 | .160 | -0.02±0.03 | -1.58 | .114 |
| Weekly work hours (0 to 40) | 0.47±0.23 | 3.88 | <.001 | 0.32±0.21 | 2.90 | .004 | 0.01±0.01 | 2.86 | .004 | 0.01±0.01 | 1.28 | .201 |
| Large (1) vs. small urban area (-1) | -1.43±4.05 | -0.70 | .486 | 0.13±3.65 | 0.07 | .943 | -0.12±0.14 | -1.74 | .082 | -0.10±0.12 | -1.60 | .111 |
| Medium-size (1) vs. small (-1) urban area | -0.96±3.83 | -0.49 | .625 | -0.60±3.45 | -0.34 | .734 | -0.03±0.13 | -0.42 | .675 | -0.01±0.12 | -0.23 | .819 |
| High (1) vs. low household income (-1) | 10.16±5.11 | 3.91 | <.001 | 4.93±4.68 | 2.07 | .039 | 0.41±0.17 | 4.61 | <.001 | 0.26±0.16 | 3.18 | .002 |
| Average (1) vs. low household income (-1) | -1.03±4.09 | -0.49 | .623 | 0.01±3.69 | 0.01 | .995 | -0.08±0.14 | -1.14 | .254 | -0.07±0.13 | -1.03 | .304 |
| Walk/biking (1) vs. public transit (-1)^c^ | 1.19±4.57 | 0.51 | .609 | 1.84±4.11 | 0.88 | .381 | -0.05±0.16 | -0.64 | .524 | -0.07±0.14 | -0.96 | 340 |
| Car (1) vs. public transit (-1) | -0.15±4.12 | 0.07 | .943 | -0.62±3.71 | 0.33 | .742 | 0.04±0.14 | 0.52 | .607 | 0.04±0.13 | 0.61 | .544 |
| Daily commute time from work (minutes)^c^ | -0.08±0.15 | -1.01 | .314 | -0.07±0.13 | -0.98 | .329 | -0.00±0.01 | -0.29 | .769 | 0.00±0.00 | 0.16 | .871 |
| STS commute from work | 12.83±3.02 | 8.36 | <.001 | 8.01±2.83 | 5.56 | <.001 | 0.38±0.12 | 7.20 | <.001 | 0.19±0.09 | 3.74 | <.001 |
| **Increment** | **∆R^2^ = .11,**  ***F*(4, 605) = 21.62, *p*<.001** | | | **∆R^2^ = .05,**  ***F*(4, 604) = 10.47, *p*<.001** | | | **∆R^2^ = .07,**  ***F*(4, 605) = 14.26, *p*<.001** | | | **∆R^2^ = .02,**  ***F*(4, 604) = 3.59, *p*<.001** | | |
| **Full model** | **R^2^= .20,**  ***F*(15, 605) = 10.28, *p*<.001** | | | **R^2^= .36,**  ***F*(16, 604) = 20.78, *p*<.001** | | | **R^2^= .21,**  ***F*(15, 605) = 10.91, *p*<.001** | | | **R^2^= .36,**  ***F*(16, 604) = 21.51, *p*<.001** | | |

Note. Missing values were excluded pairwise.

^a^An index calculated as the sum of rated frequencies (*0=never; 1=rarely; 2=sometimes; 3=often; 4=very often*) multiplied by intensities (*slightly=1; moderately=2*; *very=3*) for negative emotions (*sad, passive, depressed, sleepy, dull,* and *displeased*) subtracted from the sum of rated frequencies multiplied by intensity for positive emotions (*glad, active, joyful, awake, peppy,* and *pleased*). The index ranges from -144 to 144.

^b^Average ratings of agreement (from 1 do not agree to 7 fully agree) to the following statements: *In most ways my life is close to my ideal; The conditions of my life are excellent; I am satisfied with my life; So far I have received the important things I want in life*; *If I could live my life over, I would change almost nothing*.

^c^Significant effects on affect balance before STS was entered.
